# Surgical management and perioperative morbidity of patients with primary borderline ovarian tumor (BOT)

**DOI:** 10.1186/1757-2215-6-48

**Published:** 2013-07-09

**Authors:** Fabian Trillsch, Jan David Ruetzel, Uwe Herwig, Ulrike Doerste, Linn Woelber, Donata Grimm, Matthias Choschzick, Fritz Jaenicke, Sven Mahner

**Affiliations:** 1 Department of Gynecology and Gynecologic Oncology, University Medical Center Hamburg-Eppendorf, Martinistrasse 52, 20246 Hamburg, Germany; 2 Department Gynecology and Obstetrics, Albertinen-Krankenhaus, Hamburg, Germany; 3 Institute of Pathology, University Medical Center Hamburg-Eppendorf, Hamburg, Germany

**Keywords:** Borderline ovarian tumor, Surgical management, Complications, Morbidity, Laparoscopy, Laparotomy

## Abstract

**Background:**

Surgery is the cornerstone for clinical management of patients with borderline ovarian tumors (BOT). As these patients have an excellent overall prognosis, perioperative morbidity is the critical point for decision making when the treatment strategy is developed and the primary surgical approach is defined.

**Methods:**

Clinical and surgical parameters of patients undergoing surgery for primary BOT at our institutions between 1993 and 2008 were analyzed with regard to perioperative morbidity depending on the surgical approach (laparotomy vs. laparoscopy).

**Results:**

A total of 105 patients were analyzed (44 with primary laparoscopy [42%], 61 with primary laparotomy [58%]). Complete surgical staging was achieved in 33 patients at primary surgical approach (31.4%) frequently leading to formal indication of re-staging procedures. Tumor rupture was significantly more frequent during laparoscopy compared to laparotomy (29.5% vs. 13.1%, p = 0.038) but no other intraoperative complications were seen in laparoscopic surgery in contrast to 7 of 61 laparotomies (0% vs. 11.5%, p = 0.020). Postoperative complication rates were similar in both groups (19.7% vs. 18.2%, p = 0.848).

**Conclusions:**

Irrespective of the surgical approach, surgical management of BOT has acceptable rates of perioperative complications and morbidity. Choice of initial surgical approach can therefore be made independent of complication-concerns. As the recently published large retrospective AGO ROBOT study observed similar oncologic outcome for both approaches, laparoscopy can be considered for staging of patients with BOT if this appears feasible. An algorithm for the surgical management of BOT patients has been developed.

## Introduction

Borderline ovarian tumors (BOT) are epithelial tumors constituting for approximately 10-20% of all ovarian neoplasias [1]. As the term borderline implies they clinically behave intermediate between benign and malignant tumors. Histologically this is expressed by cellular proliferation and nuclear atypia without destructive stromal invasion [2]. BOT have an excellent overall prognosis with a 5-year-survival rate of almost 90% across all stages but frequently recur within the abdomen even after more than ten years [3,4].

Current guidelines recommend similar surgical management of BOT patients compared to ovarian cancer patients except for lymphadenectomy [5]. Fertility sparing surgery can be performed when the uterus and at least part of one ovary are preserved leading to slightly elevated but acceptable recurrence rates [1,6]. The majority of BOT patients are diagnosed in early stage when the disease is still limited to the ovaries (78.5% in FIGO stage IA/B) [7]. However, the diagnosis of BOT is usually not established prior to or during surgery due to nonspecific clinical and diagnostic features as well as limited diagnostic accuracy of intraoperative histology. Therefore many patients require re-staging surgery, even though the prognostic indication for this procedure remains controversial [8].

The surgical approach for BOT is still under debate. As laparoscopy became standard approach for benign ovarian lesions [9] it has not convincingly proven to guarantee adequate staging and oncologic safety in BOT patients [10]. Taking the excellent overall survival and low recurrence rates into account, the degree of perioperative morbidity is an important aspect when counselling BOT patients. We therefore systematically assessed BOT patients of our institutions focusing on surgical parameters and complications to evaluate the optimal surgical approach.

## Methods

### Patients

All patients with primary diagnosis of BOT who presented for surgery at the University Medical Center Hamburg-Eppendorf and the Albertinen-Krankenhaus Hamburg between 1993 and 2008 were retrospectively investigated. Of 105 analyzed patients, 95 were part of the retrospective multicenter outcome survey of AGO-Ovarian Cancer Study Group (ROBOT; AGO-OVAR OP.5) which has been recently published [11]. Primary surgery was defined as the procedure in which the diagnosis of BOT was first established irrespective of the surgical extend. Depending on preoperative clinical features of the initial diagnostic workup with gynecologic examination including vaginal ultrasound and the surgeon’s surgical skills and preference, the surgical approach was determined (e.g. tumor size, aspect in ultrasound, presence of ascites, comorbidities, BMI, CA125 level). Additional radiologic examinations were performed if necessary. Solely definite histology was accepted to establish the diagnosis of BOT but intraoperative histology was frequently applied to adjust the surgical extend during primary surgery.

Complete surgical staging was defined as bilateral salpingoophorectomy, omentectomy, peritoneal washing and biopsies as well as appendectomy for mucinous histology. Fertility sparing staging was discussed with patients of reproductive age to preserve the uterus and at least a part of one ovary. A thorough exploration of the abdomen was part of each surgery. Re-staging surgery of BOT patients was defined to be a secondary surgery in which parts of the recommended staging were completed. All patients received prophylaxis for thromboembolism with low-molecular weight heparin (Enoxaparin-Natrium 20 mg) from the day of surgery until at least the day of discharge from hospital.

Data was retrieved from patients’ records and the tumor registry. Written informed consent was obtained from all patients to access their tissue and review their medical records when they first attended the hospital according to our investigational review board and ethics committee guidelines (reference number #190504). All pathological studies were performed by specialized gynecopathologists in the department of pathology of the University Medical Center Hamburg-Eppendorf. Pathological reports were retrospectively analyzed for tumor classification, stage, histological subtype, implants and other prognostic factors such as microinvasion and micropapillary structure. The tumor staging classification of the Fédération Internationale de Gynécologie et d’Obstétrique (FIGO) [12] was used to determine the stage of disease.

### Analysis of surgical procedures

To analyze the characteristics of the primary surgical treatment, surgery reports of BOT patients were systematically reviewed for localization of the tumor, performed procedures, the need for conversion from laparoscopy to laparotomy, occurrence of tumor cyst rupture, duration of surgery and the result of surgical resection (complete vs. incomplete). The same characteristics were analyzed for all re-staging procedures.

To evaluate perioperative morbidity, intraoperative as well as postoperative complications were analyzed. Inhospital stay and the required days of intensive care were noted. Intraoperative events as transfusion of blood products, cardiac events, bladder or ureter lesions, bowel lesions and lesions of other abdominal organs were retrieved from the surgical reports and anesthesiological protocols. For the postoperative course, medical charts were analyzed for the number of transfused blood products as well as for postoperative hemorrhage, infections, septicemia, temperature >38.5°C after surgery, fever >6 days after surgery, blood glucose dysregulation, delayed wound healing or wound breakdown, symptomatic lymphatic cysts, cardiac and circulatory events, deep vein thrombosis, postoperative pneumothorax or pleural effusion, pulmonary embolism, urinary retention, renal failure, subileus, ileus and the need for re-laparotomy due to complications.

### Statistical analysis

All statistical analyses were conducted with SPSS software version 19.0 (SPSS Inc., Chicago, IL, USA). *Mann–Whitney U test* was applied as non-parametric significance test. In addition, *Chi-quadrate (Chi*^*2*^*) test* and *Fisher’s exact test* were performed to analyze the difference in patient characteristics, surgical parameters and complications for each surgical approach. P-values less than 0.05 were regarded as statistically significant.

## Results

### BOT patients and primary surgery

A total of 105 patients were analyzed in this study. For 61 cases (58.1%) the primary surgical approach was by laparotomy, 44 patients (41.9%) initially underwent laparoscopy. Detailed patient characteristics are listed in Table 1. Patients in the laparotomy group were significantly older with a median age of 59 years (range 13–85 years) compared to 47 years (range 16–86 years) in the laparoscopy group (p = 0.006). Especially patients in the reproductive age (< 40 years) were more likely to receive primary laparoscopy (36.4% vs. 16.4%, p = 0.023).

**Table 1:** Patient characteristics

	**Primary approach via laparotomy**		**Primary laparoscopic approach**		**P**
	**(n = 61)**	**(58.1%)**	**(n = 44)**	**(41.9%)**	
**Age, median (years)**	**59**		**47**		**0.006**^**a**^
*range*	*13-85*		*16-86*		
≤40	10	16.4%	16	36.4%	**0.023**^**c**^
41-55	18	29.5%	16	36.4%	0.528^c^
56-65	12	19.7%	5	11.4%	0.294^c^
>65	21	34.4%	7	15.9%	**0.044**^**c**^
**FIGO stage**					
I A/B	40	65.6%	19	43.2%	**0.029**^**c**^
I C	13	21.3%	19	43.2%	**0.019**^**c**^
II A-C	1	1.6%	2	4.5%	0.570^c^
III A/B	3	4.9%	3	6.8%	0.693^c^
III C	4	6.6%	1	2.3%	0.396^c^
IV	0	0.0%	0	0.0%	
**Histologic subtype**					
serous	27	44.3%	32	72.7%	**0.005**^**c**^
seromucinous	2	3.3%	5	11.4%	0.127^c^
mucinous	30	49.2%	6	13.6%	**< 0.001**^**c**^
endometrioid	2	3.3%	1	2.3%	1.000^c^
**Implants**					
yes	8	13.1%	2	4.5%	0.140^b^
*- invasive*	*1*		*0*		
*- non-invasive*	*7*		*2*		
no	53	86.9%	42	95.5%	
**Ascites**					
none	27	44.3%	18	40.9%	0.468^b^
< 500 ml	22	36.1%	11	25.0%	
> 500 ml	8	13.1%	2	4.5%	
unknown	4	6.6%	13	29.5%	
**CA 125**	**44.6**			**48.5**	0.804^a^
*range*	*2-2770*			*6-360*	
elevated	27	44.3%	16	36.4%	0.594^b^
within normal limits	24	39.3%	11	25.0%	
unknown	10	16.4%	17	38.6%	
**BMI, median**	**23,4**		**24.1**		0.904^a^
*range*	*17,7-43,0*		*18.6-35.3*	*17,7-43,0*	
< 19	3	4.9%	1	2.3%	0.677^b^
19-24	23	37.7%	15	34.1%	
24-30	19	31.1%	11	25.0%	
>30	8	13.1%	2	4.5%	
unknown	8	13.1%	15	34.1%	
**ASA, median**	**2**		**2**		**0.010**^**a**^
*range*	*1-3*		*1-3*	*1-3*	
1	7	11.5%	13	29.5%	**0.020**^**b**^
2	31	50.8%	14	31.8%	
3	9	14.8%	3	6.8%	
unknown	14	23.0%	14	31.8%	

Overview of the characteristics of all BOT patients (n = 105). Sixty-one patients undergoing primary laparotomy were opposed to 44 patients receiving primary laparoscopy.

*(*^*a*^*: Mann–Whitney U test,*^*b*^*: Chi*^*2*^*test,*^*c*^*: Fisher’s exact test).*

The vast majority of patients underwent surgery because of suspicious ovarian masses (93.3%), in seven cases (6.7%) the diagnosis of BOT was established unexpectedly during surgery for different reasons (e.g. Caesarean section, suspected appendicitis, endometriosis, ovarian torsion). Most patients (59 of 105, 56.2%) were treated in an early stage when the disease was still limited to the ovaries (FIGO stage IA/B). Thirteen patients were diagnosed and treated with tumor mass extending the ovaries (13.3%, FIGO stage ≥ IIA). Mucinous histology was more frequently seen in laparotomies (49.2% vs. 13.6% in laparoscopies, p < 0.001) as these tumors were more associated with the presence of ascites than other histologies. Peritoneal implants could be detected in ten patients (9.5%), only in one case the implants showed an invasive growth pattern. Forty-three of all BOT patients (41.0%) had ascites, of them ten patients (23.3%) with a volume of >500 ml but without significant impact on the decision of the surgical approach.

During initial surgery complete tumor resection was achieved in 90 of all patients (84.8%) and in 66 cases intraoperative histology was obtained (62.9%). This rate was significantly higher in laparotomies (83.6%) with 30 consecutive intraoperative diagnoses of BOT compared to 34.1% and nine intraoperative diagnoses of BOT in primary laparoscopies (p < 0.001). Only 33 of all patients (31.4%) underwent complete staging procedures during primary surgery. Of these, 29 were performed by laparotomy (47.5% of all laparotomies). In contrast, 4 of the 44 primary laparoscopic surgeries led to a complete surgical staging (9.1%, p < 0.001). These rates equalized after re-staging procedures with complete surgical staging in 50.8% of all laparotomies and 52.3% of all laparoscopies.

### Surgical morbidity

In the group of 44 patients undergoing primary laparoscopy, the surgical approach had to be converted to laparotomy in 14 patients (31.8%). In three of these cases (21.4%) the conversion was already planned preoperatively in case of intraoperative diagnosis of BOT or ovarian cancer. Eight approaches were converted due to disseminated tumor growth pattern or tumor size and three due to adhesions, not manageable by laparoscopy. A detailed overview of the surgical parameters is given in Table 2. Median operating time did not significantly differ (112 min vs. 136 min, p = 0.120) but patients undergoing laparoscopy had a significantly shorter median inhospital stay for primary surgery (5 days vs. 9 days, p < 0.001). Cyst rupture of the primary tumor occurred in 21 cases (20.0%) which was significantly less frequent during laparotomy (13.1%) compared to laparoscopy (29.5%, respectively, p = 0.038). Sixteen of all patients (15.2%) were monitored on intermediate or intensive care units postoperatively. For the majority of patients (91.4%) no blood products had to be transfused perioperatively, although median postoperative haemoglobin levels were significantly lower compared to preoperative levels (11.2 vs. 13.3 mg/dL, p < 0.001), favouring laparoscopy over laparotomy (11.9 vs.10.9 mg/dL, p = 0.006).

**Table 2:** Surgical parameters

	**Primary approach via laparotomy**		**Primary laparoscopic approach**		**P**
	**(n = 61)**	**(58.1%)**	**(n = 44)**	**(41.9%)**	
**Indication for surgery**					
cystic ovarian lesion	56	91.8%	42	95.5%	
*- unsuspicious ovarian cyst*	*0*		*13*		
*- cystic lesion of unknown dignity*	*48*		*29*		
*- suspected ovarian cancer/ BOT*	*8*		*0*		
unexpected diagnosis during surgery for different reason	5	8.2%	2	4.5%	
**Conversion of laparoscopy to laparotomy***(reason for conversion)*			14	31.8%	
intraoperative conditions			11	78.6%	
*- tumor growth pattern and tumor size*			*8*		
*- adhesions*			*3*		
result of intraoperative histology			3	21.4%	
**Surgical procedures in primary and re-staging surgery**					
fertility sparing surgery	9	14.8%	15	34.1%	
non-fertility sparing surgery	52	85.2%	29	65.9%	
omentectomy	33	54.1%	30	68.2%	
appendectomy	35	57.4%	12	27.3%	
peritoneal biopsies	37	60.7%	23	52.3%	
cytology	49	80.3%	38	86.4%	
sampling of lymph nodes or lymphonodectomy	24	39.3%	14	31.8%	
*- pelvic*	*19*		*13*		
*- paraaortic*	*5*		*3*		
**Surgical cytoreduction**					0.333^b^
complete	54	88.5%	36	81.8%	
incomplete	7	11.5%	8	18.2%	
**Surgical staging**					**< 0.001**^**b**^
- complete in primary surgery	30	49.2%	4	9.1%	
- incomplete in primary surgery	31	50.8%	40	90.9%	
- complete after re-staging surgery	31	50.8	23	52.3	
- incomplete after re-staging	30	49.2	21	47.7	
**Intraoperative histology**					**< 0.001**^**b**^
obtained	51	83.6%	15	34.1%	
*- intraoperative diagnosis of BOT*	*30*		*9*		
not obtained	10	16.4%	29	65.9%	
**Cyst rupture**					**0.038**^**b**^
yes	8	13.1%	13	29.5%	
no	53	86.9%	31	70.5%	
**Duration of surgery, median (min)**	136		112		0.120^a^
*range*	*50-405*		*47-350*		
**Eks, median**	0		0		0.088^a^
*range*	*0-6*		*0-2*		
**FFPs, median**	0		0		0.342^a^
*range*	*0-20*		*0-10*		
**Hb preop, median (mg/dl)**	13.4		13.6		0.662^a^
*range*	*5.6-15.1*		*10.3-14.8*		
**Hb postop, median (mg/dl)**	10.9		11.9		**0.006**^**a**^
range	7.4-13.6		9.5-14.3		
**Days on ICU, median (days)**	0		0		**0.014**^**a**^
*range*	*0-4*		*0-2*		
**Primary inhospital stay, median (days)**	9		5		**< 0.001**^**a**^
*range*	*5-19*		*0-19*		

Detailed overview of the surgical procedures as well as the characteristics of surgery and the inhospital stay. Parameters were separately analyzed for laparotomy (n = 61) and laparoscopy (n = 44).

*(*^*a*^*: Mann–Whitney U test,*^*b*^*: Chi*^*2*^*test).*

**Figure 1:**
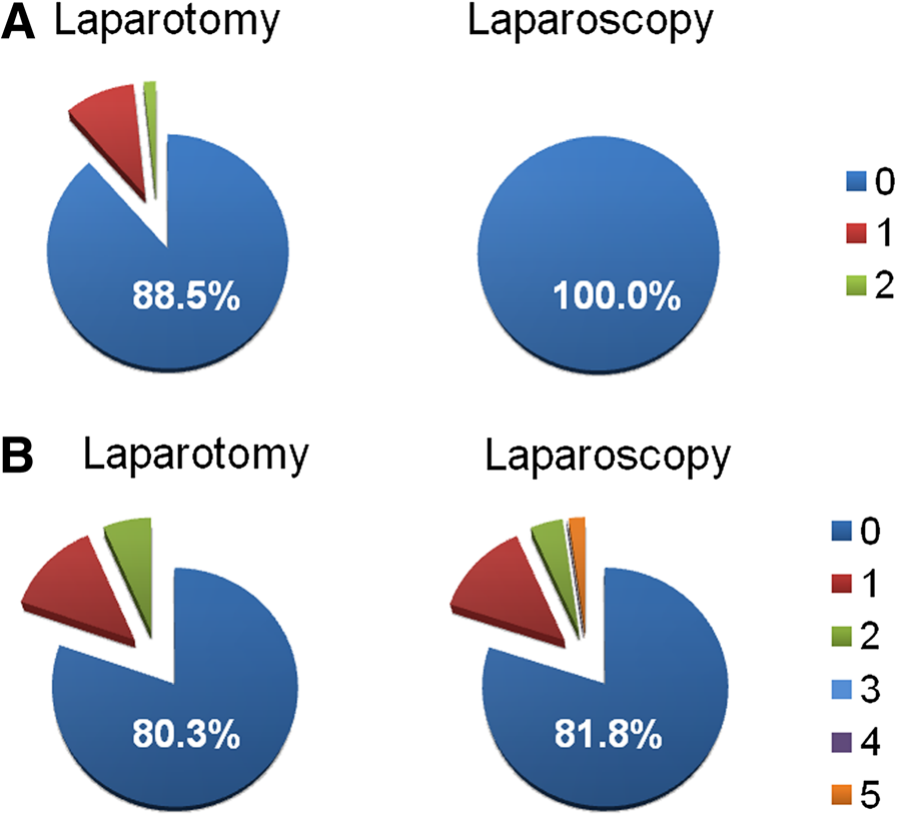
**Perioperative complications. A**. Number of intraoperative complications. **B**. Number of postoperative complications. The number of perioperative complications (1–5) of patients undergoing surgery for BOT is visualized for primary laparotomy or laparoscopy, respectively. The percentage of patients without complications is mentioned in each diagram.

There was a trend for higher perioperative complication rates in laparotomies compared to laparoscopies (29.5% vs. 18.2%, p = 0.185) although this finding did not reach statistical significance. A detailed list of these events is shown in Table 3 and Figure 1 revealing that this difference is result of the intraoperative complication rates. During primary laparoscopy no complication occurred intraoperatively, while eight patients experienced postoperative complications (18.2%). In contrast, intraoperative complications occurred in 7 of 61 laparotomies (11.5% vs. 0%, p = 0.020) and postoperative complications were noted in 20 patients (19.7% vs. 18.2%, p = 0.848). An intermittent cardiac arrest and a bladder lesion occurred during primary laparotomy in one patient, in another surgery the spleen had to be removed due to intraoperative lesion. Five patients received blood transfusions intraoperatively. Transfusion of red blood cells was also the most frequent postoperative event after laparotomy with five patients in this group. More serious postoperative complications included deep vein thrombosis after laparotomy as well as acute renal failure and ileus after laparoscopy. The rate for wound infection and delayed fever showed a trend for higher rates in laparoscopies compared to laparotomies (16.7% vs. 5.9% and 16.7% vs. 11.8%) but without reaching statistical significance. Of note, three of these four laparoscopic cases were observed in laparoscopies intraoperatively converted to laparotomy.

**Table 3:** Perioperative complications

	**Primary approach via laparotomy**		**Primary laparoscopic approach**		**P**	** All surgeries**	
	**61**		**44**			**105**	
**Overall complications**							
**No complications**	**43**	**70.5%**	**36**	**81.8%**	0.185^b^	79	**75.2%**
**Yes**	**18**	**29.5%**	**8**	**18.2%**		26	**24.8%**
**Intraoperative complications**							
**No complications**	**54**	**88.5%**	**44**	**100.0%**	**0.020**^**b**^	98	**93.3%**
**Yes**	**7**	**11.5%**	**0**	**0.0%**		7	**6.7%**
*Intraoperative transfusions*	*5*	*62.5%*	*0*			*5*	*62.5%*
*Cardiac arrest*	*1*	*12.5%*	*0*			*1*	*12.5%*
*Bladder lesions*	*1*	*12.5%*	*0*			*1*	*12.5%*
*Ureter lesions*	*0*	*0%*	*0*			*0*	*0%*
*Bowel lesions*	*0*	*0%*	*0*			*0*	*0%*
*Intraoperative splenic injury*	*1*	*12.5%*	*0*			*1*	*12.5%*
**Complications in total**	**8**	**100.0%**	**0**			**8**	**100.0%**
**Postoperative complications/morbidity**							
**No complications**	**49**	**80.3%**	**36**	**81.8%**	0.848^b^	85	**81.0%**
**Yes**	**12**	**19.7%**	**8**	**18.2%**		20	**19.0%**
*Postoperative transfusion*	*5*	*29.4%*	*1*	*8.3%*		*6*	*20.7%*
*Postoperative hemorrhage*	*0*	*0%*	*0*	*0%*		*0*	*0%*
*Blood glucose dysregulation*	*1*	*5.9%*	*0*	*0%*		*1*	*3.4%*
*Fever >6 days after surgery*	*1*	*5.9%*	*2*	*16.7%*		*3*	*10.3%*
*Wound infection*	*2*	*11.8%*	*2*	*16.7%*		*4*	*13.8%*
*Delayed wound healing*	*1*	*5.9%*	*1*	*8.3%*		*2*	*6.9%*
*Persistent lymphatic cysts*	*1*	*5.9%*	*0*	*0%*		*1*	*3.4%*
*Circulatory dysregulation*	*1*	*5.9%*	*2*	*16.7%*		*3*	*10.3%*
*Deep vein thrombosis*	*2*	*11.8%*	*0*	*0%*		*2*	*6.9%*
*Pulmonary embolism*	*0*	*0%*	*0*	*0%*		*0*	*0%*
*Acute renal failure*	*0*	*0%*	*1*	*8.3%*		*1*	*3.4%*
*Urinary retention*	*1*	*5.9%*	*0*	*0%*		*1*	*3.4%*
*Ileus*	*0*	*0%*	*1*	*8.3%*		*1*	*3.4%*
*Pleural effusion*	*0*	*0%*	*1*	*8.3%*		*1*	*3.4%*
*Pain syndrome*	*1*	*5.9%*	*0*	*0%*		*1*	*3.4%*
*Re-Laparotomy*	*0*	*0%*	*2*	*16.7%*		*2*	*6.9%*
**Complications in total**	**16**	**100.0%**	**13**	**100.0%**		**29**	**100.0%**

Complications of primary laparotomy (n = 61) and laparoscopy (n = 44), multiple complications per patient possible. Besides overall complication rates, intraoperative and postoperative events are listed separately.

*(*^*b*^*: Chi*^*2*^*test).*

Re-laparotomy had to be performed in two patients of the laparoscopy group. In one patient, extensive wound infection following laparoscopy with conversion to laparotomy caused re-laparotomy while the other patient experienced a postoperative mechanical ileus due to adhesions after laparoscopic surgery. Except for one patient, all patients with perioperative complications had less than three events during their inhospital stay. For the other patient undergoing primary laparoscopy converted to laparotomy, the postoperative course was complicated by five events in total. Besides postoperative transfusions and an acute renal failure, the patient underwent re-laparotomy due to the extensive wound infection in which a pleural effusion was intraoperatively drained.

## Discussion

The importance of complete surgical staging and the optimal surgical approach to achieve this aim have to be discussed with borderline ovarian tumor (BOT) patients during initial consultation and while the treatment strategy is being developed.

In this report, we analyzed 105 BOT patients surgically treated at the University Medical Center Hamburg-Eppendorf and the Albertinen-Krankenhaus Hamburg to determine perioperative morbidity depending on the surgical approach. Forty-four patients initially receiving laparoscopic surgery were opposed to 61 patients with primary approach by laparotomy. Of note, patients in this study were retrospectively included and not randomly assigned to the surgical approach which warrants cautious interpretation due to possible selection bias. However, owing to the bi-centric nature of our study, all patients were treated with comparable surgical standards, received a thorough perioperative workup and detailed documentation. Surgeries were performed by a specialized and experienced team of gynecologic oncologists.

In contrast to benign lesions, where it is widely accepted to perform laparoscopy for adnectomy or cystectomy [9] no clear selection criteria exist for BOT patients to define the primary surgical approach [10]. Patients with ovarian cancer require laparotomy to guarantee best conditions for a thorough exploration, tumor resection and staging procedures [13]. In BOT patients, laparoscopy appears to be an alternative as these tumors are usually diagnosed in early stage when the disease is limited to the ovaries and lymphadenectomy is not included in current recommendations for surgical staging [5,7,14]. A major concern regarding laparoscopy are higher rates of cyst rupture consecutively leading to an iatrogenic spread of tumor cells with possible impact on the prognosis and clinical course of the patients [10]. This is also reflected in our study where a significant difference in rates for cyst rupture was seen, resulting in a significantly higher rate of FIGO stage IC patients in the laparoscopy group. Consequences for prognosis were not evaluated in our analysis. However, previous studies could not demonstrate significant difference in recurrence rates for these patients when the two surgical approaches were compared which was recently confirmed by the so far largest, retrospective multicenter outcome survey of AGO-Ovarian Cancer Study Group (ROBOT; AGO-OVAR OP.5 [10,11]). Potential prognostic consequence must, nevertheless, be considered when advantages of laparoscopic surgeries as shorter inhospital stay, smaller wound surface and earlier postoperative mobilisation are discussed with the patients.

At present, the decision on the surgical approach for BOT patients is based on preoperative diagnostic features, epidemiological aspects and the surgeon’s preference. In our cohort, patients receiving laparoscopy were significantly younger and had a significantly better ASA performance status. A suspicious ovarian lesion was the indication for laparoscopy in the majority of our patients. Only one third of these patients received intraoperative frozen section analysis underlining that the intention of most laparoscopies was more diagnostic than achieving a definite surgical therapy. As a consequence, the rate of complete tumor resection was lower and re-staging surgery was formally indicated in the majority of cases. Fourteen of all 44 laparoscopies (31.8%) had to be converted to laparotomy which correlates with other studies reporting of approximately 30% conversion rates for BOT patients [10]. The majority of conversions were result of adhesions, disseminated tumor growth pattern or tumor size.

Intraoperative complications were generally low in our cohort with an overall rate of 6.7%. No intraoperative event was registered in primary laparoscopies while in seven laparotomies complications occurred (11.5%). Only one patient required mass transfusions with six packed red blood cells and 20 fresh frozen plasmas during a complicated laparotomy for an advanced stage mucinous BOT. To put these rates in context, it is important to note that the intention and the surgical extend varied substantially. The rate of complete surgical staging was significantly higher in the laparotomy group so that these patients underwent more advanced surgical steps as hysterectomy and tumor debulking with consecutively rising risk for complications. Therefore, the significant difference in complications has to be regarded cautiously and should not lead to consequences in management alone.

Interestingly, postoperative morbidity and complications in the present study were comparable irrespective of the surgical approach (19.7% for laparotomy vs. 18.2% for laparoscopy). For all patients, postoperative hemoglobin levels were significantly lower compared to preoperative levels, but blood transfusions were only needed in six patients during the postoperative course. This was significantly more frequent following laparotomy while the rate of wound infections and delayed fever tended to be higher in the laparoscopy group. However, this was not statistically significant and three of four cases with postoperative infections occurred after laparoscopies converted to laparotomies. Most serious events in the overall cohort were two deep vein thromboses, one patient with acute renal failure and ileus requiring surgical revision.

To the best of our knowledge, there are no reports available focusing on complication rates of BOT patients so that a direct comparison of our cohort and published series is not possible. For primary ovarian cancer, perioperative complication rates from 8 to 58% have been described [15-17]. In a previous study of our group concentrating on advanced stage ovarian cancer, we observed 36.0% perioperative complications rates after radical surgical treatment [17]. In contrast to our BOT patients, all of these surgeries were performed by laparotomy, the majority of the patients had advanced stage disease (94.3% FIGO stage IIIC/IV ovarian cancer) requiring radical procedures as bowel resection in 62.1% of cases or systematic pelvic and paraaortic lymphadenectomy in 73.0%. This underlines that the principal surgical extend for BOT patients compared to ovarian cancer patients is different.

**Figure 2:**
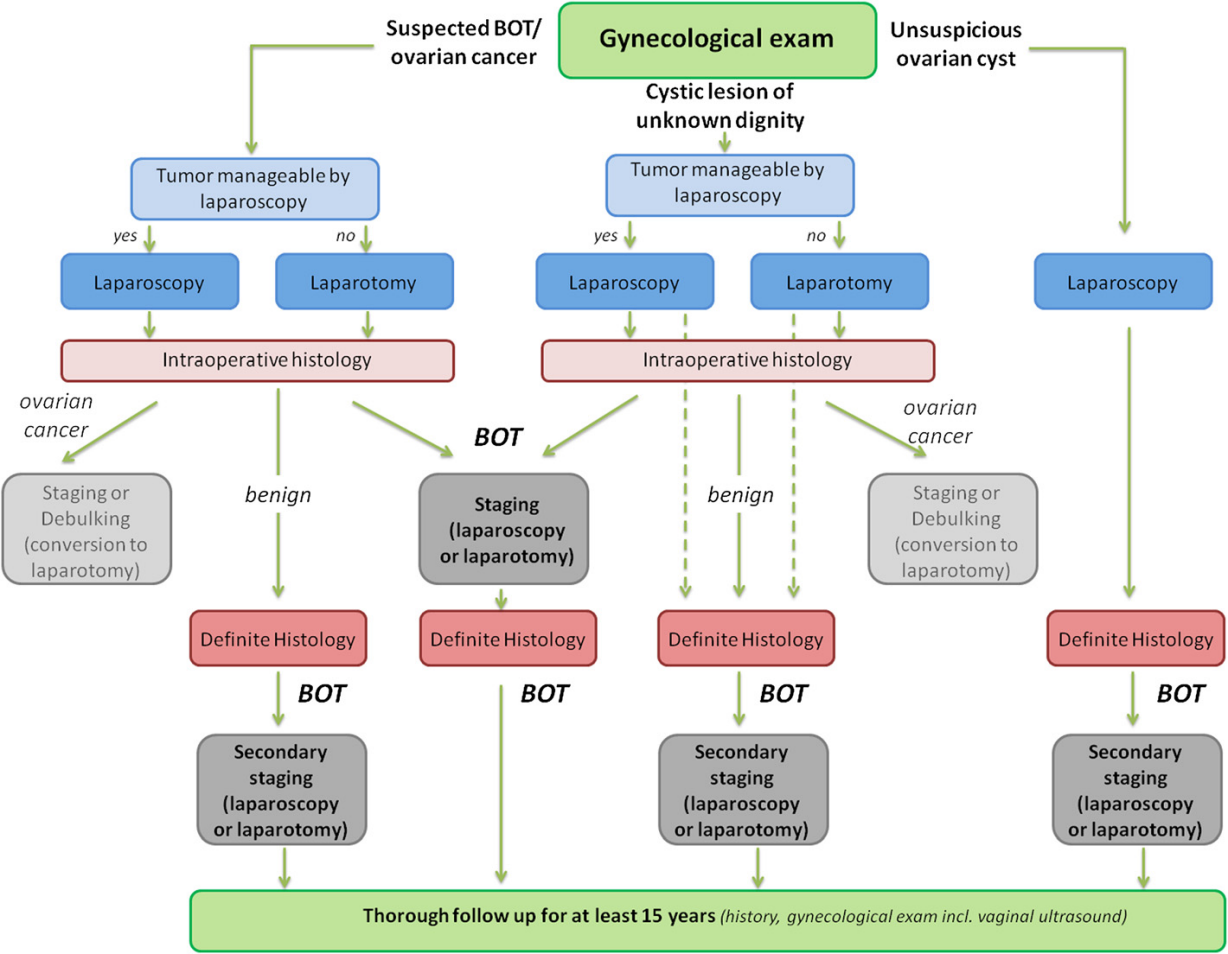
Algorithm for surgical management of BOT patients.

We therefore regard this first report specifically concentrating on surgical parameters and perioperative morbidity of BOT patients as an important basis for further, preferably multicentric analyses. From our perspective a one-timed approach should be aimed applying intraoperative fresh frozen section analysis to adjust the surgical extend. However, the accuracy of intraoperative diagnosis for BOT is rather low and postponing the definite surgical management to a completion surgery appears to be an inevitable alternative [14,18]. After diagnosis of BOT intraoperatively or in definite histology complete surgical staging has to be performed. Based on the initial gynecological examination the surgical approach has to be defined by the surgeon. In case of unsuspicious ovarian lesions laparoscopy is preferred, for cystic tumors of unknown dignity or suspected BOT laparoscopy can be considered if tumor appears manageable by laparoscopy and there is no evidence for abdominal tumor dissemination. We developed a treatment algorithm guiding the gynecologic oncologist through the decision making process (see algorithm Figure 2). The present results might therefore help gynecologic oncologists to put surgical management and perioperative morbidity into perspective.

## Conclusions

In the present study of 105 BOT patients, we could demonstrate that surgical treatment has acceptable complication rates and perioperative morbidity irrespective of the surgical approach.

As patients in this study were not randomly assigned to primary laparoscopic or open surgical approach, differences regarding the primary indication for surgery and intraoperative procedures may influence the results. Future prospective trials are therefore highly desirable to allow evidence based recommendations for the surgical approach and a concise appraisal of surgical morbidity of BOT patients.

Until then, choice of initial surgical approach can primarily be made independent of complication-concerns. As the recently published large retrospective AGO ROBOT study observed similar oncologic outcome for both approaches, laparoscopy is preferred for unsuspicous ovarian lesions and can be considered for surgical staging in case of localized tumor and feasible intraoperative conditions.

## Competing interests

All authors declare that they do not have any financial or personal relationship with other people or organizations that could inappropriately influence this work.

## Authors’ contributions

FT and SM were responsible for the concept and design of the presented study, analyzed the data and prepared the manuscript. JDR, UH and FT collected main parts of the data, participated in the study design and supported the statistical analysis. MC performed the histopathological analyses leading to the diagnosis of BOT. All authors were involved in the data acquisition and quality control for the trial. All authors supported the manuscript editing, read and approved the final manuscript.
